# A Series of Cerebral Venous Sinus Thromboses Treated with Intra-Arterial tPA infused over Ten Hours with a 0.027-inch Catheter and Literature Review

**DOI:** 10.7759/cureus.654

**Published:** 2016-06-23

**Authors:** Endrit Ziu, O'Hara Haley, Muhammad Ibrahimi, Sara Langan, Scott Simon

**Affiliations:** 1 Department of Neurosurgery, Penn State Hershey Medical Center; 2 Neural and Behavioral Science, Penn State Hershey Medical Center; 3 Neurology, Penn State Hershey Medical Center

**Keywords:** cerebral venous sinus thrombosis, thrombolytic infusion

## Abstract

Cerebral venous sinus thrombosis (CVST) can have devastating results, with mortality reported in 44% of cases. No randomized trials exist in order to define what qualifies as failure of conservative therapy, and there is no specific intervention to date which is considered safe and effective. Case series suggest that thrombolysis infusion is safer than thrombectomy, but methods of administration, dose, and duration of therapy tend to vary widely. We present three consecutive CVST patients treated with heparin who suffered both clinical and radiographic deterioration, and went on to have endovascular therapy. Each patient was successfully recanalized by placing a 0.027-inch microcatheter at the proximal portion of the thrombus and infusing 20 mg of alteplase dissolved in 1 liter of normal saline infused at 100 ml per hour for an infusion of 2 mg of alteplase per hour for ten hours.

## Introduction

Mortality and morbidity associated with cerebral venous sinus thrombosis (CVST) have been reported to be 10% and 44%, respectively [1]. Randomized controlled trials are lacking in order to define the failure of conservative therapy. Furthermore, there is a paucity of information that demonstrates endovascular therapy is efficacious, and if so, when it should be initiated and which intervention is best. Case series suggest that thrombolysis infusion is safer than thrombectomy, but methods of administration, dose, and duration of therapy vary widely [1-2]. With such a limited evidence base, as often happens within the field of neurosurgery, practitioners are forced to make critical treatment decisions based on limited data. We have faced this exact scenario in our practice; existing literature provides only a selection of case studies describing individual techniques, unhelpful in guiding therapy or in standardizing treatment.

Here we reviewed the literature and present three of our cases. All patients agreed to participate and were explained the nature and objectives of this study, and informed consent was formally obtained. No reference to the patients' identities was made at any stage during data analysis or in the report. We emphasize treatment pitfalls as well as our positive results. We hope to provide more specific clinical guidance for surgeons faced with CVST refractory for medical management. Furthermore, we hope this review and technical notes will spur further discussion and perhaps begin the process of creating a consensus among neuroendovascular surgeons who treat this condition.

## Technical report

### Case 1

**Figure 1:**
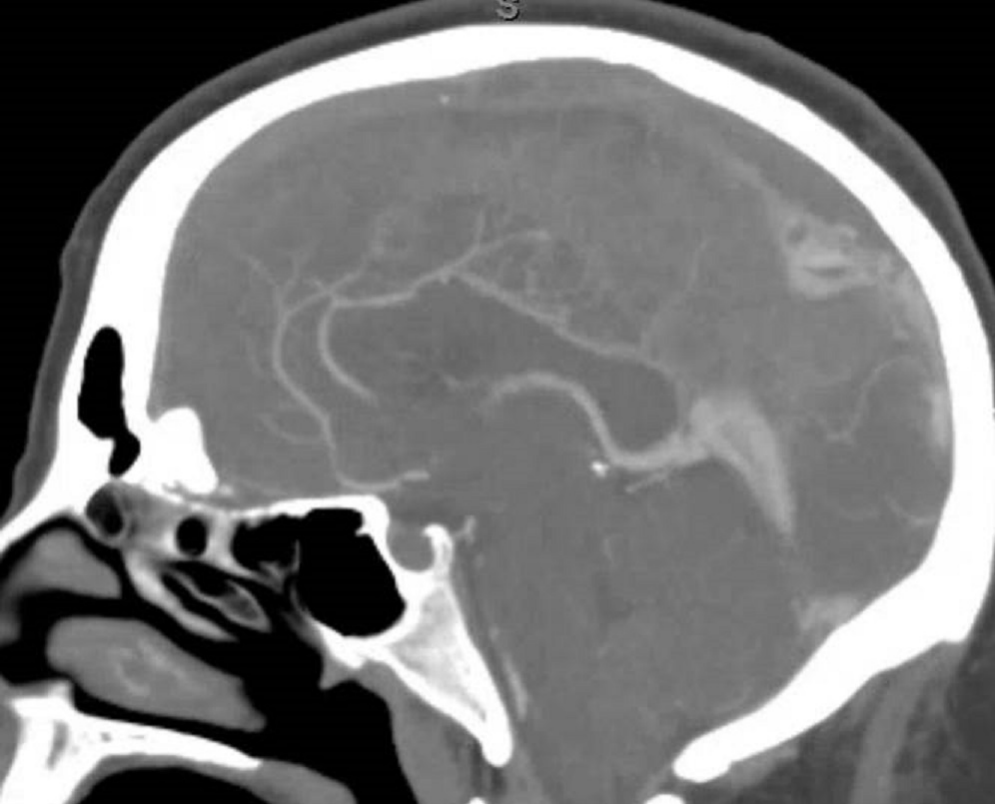
CT Angiogram at Presentation CT angiogram obtained at presentation in the sagittal view reveals no flow through sagittal sinus and flow through internal cerebral veins.

The first patient is a 30-year-old female less than one month post-partum who presented with rapid onset obtundation. MRI/V revealed superior sagittal sinus thrombosis with venous infarctions in both frontal lobes. The patient was started on a heparin drip. Her exam declined while on heparin, and she required intubation. A CT scan demonstrated increased cerebral edema as well as progression of the previously identified thrombus into the right transverse sinus (Figure 1). She was brought to angiography for possible intervention secondary to failed medical management. This confirmed that the occlusion had spread to the right transverse sinus. Both stent retriever (Solitaire FR, Medtronic) and aspiration (ACE, Penumbra, CA) were attempted without success. A 0.010-inch microcatheter (Eschelon 10, Medtronic) was placed in the anterior third of the superior sagittal sinus. The bolus dose of IV tPA was calculated to be 10 mg. This was diluted in a 500 ml bag of heparinized saline and set up for a ten-hour infusion at 100 ml per hour for a dose of 1 mg/hr in 50 ml/hr of fluid. We chose this dose after reviewing Rahman et al., and picking a dose on the low side of the spectrum provided. Unfortunately, the catheter became clogged after only three hours. 

**Figure 2:**
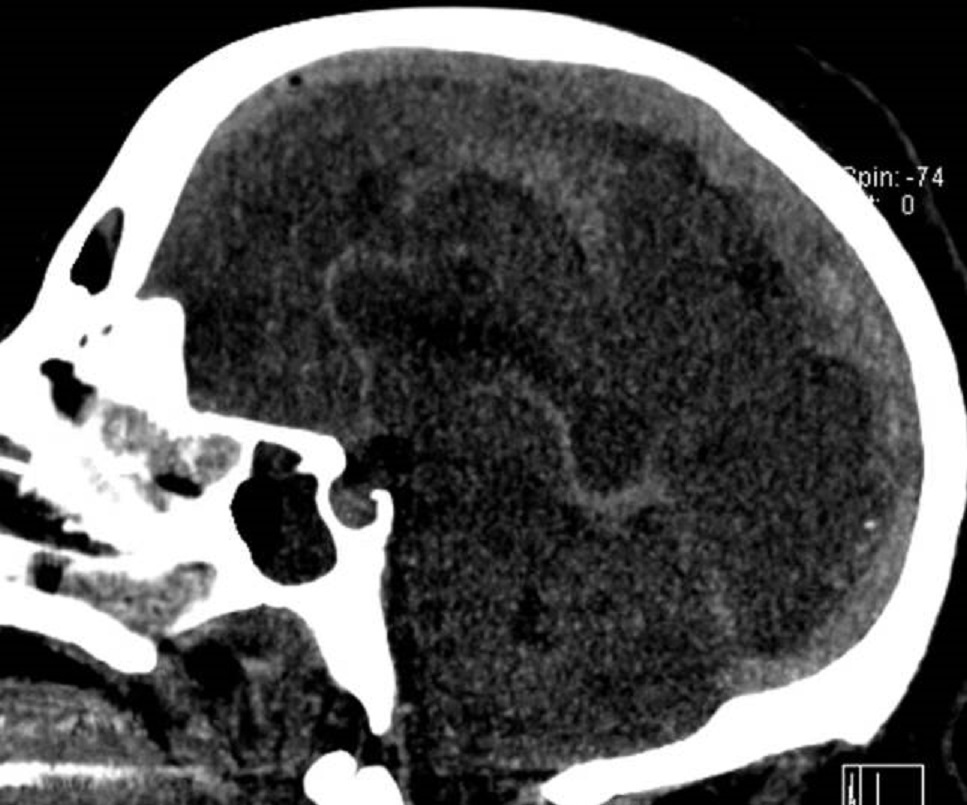
CT Venogram Post Second Infusion CT venogram obtained after second infusion with larger catheter, increased tPA dose, and larger volume of infusion reveals flow through sagittal sinus and flow through internal cerebral veins. CT also reveals air resulting from infusion.

She was taken back to angiography where persistent occlusion was demonstrated. To prevent further clogging of the catheter, the 0.010-inch microcatheter was swapped out for a larger 0.027-inch microcatheter (Marksman, Medtronic). The tPA dose was doubled to 20 mg and dissolved in a 1 liter bag of heparinized saline. The infusion was then 2 mg/hr in 100 ml/hr of fluid. This infused the entire ten hours without clogging. CTA revealed a recanalized sinus still with thrombus and no new hemorrhages (Figure 2). Heparin was then restarted. On discharge one month later, she was neurologically intact.

### Case 2

**Figure 3:**
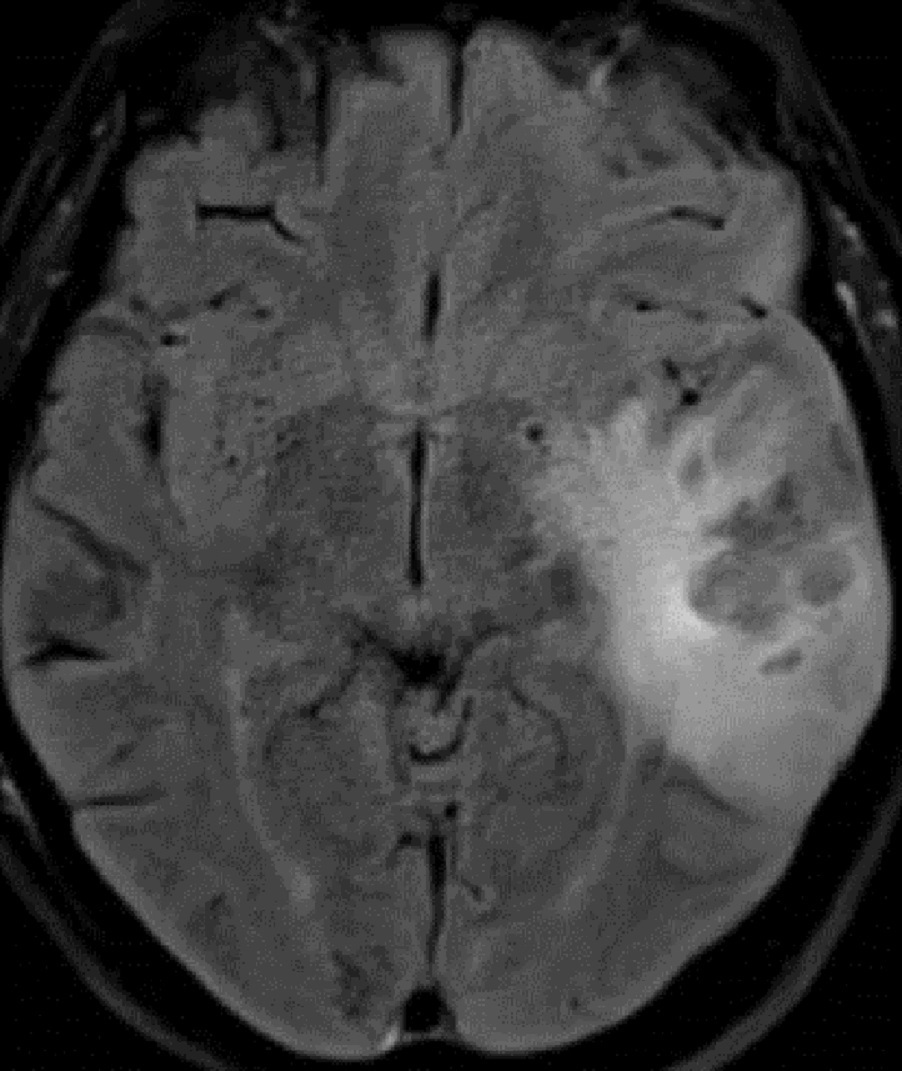
T2 MRI at Presentation T2 MRI in the axial plane obtained at presentation demonstrates classic venous infarct in the left temporal lobe.

**Figure 4:**
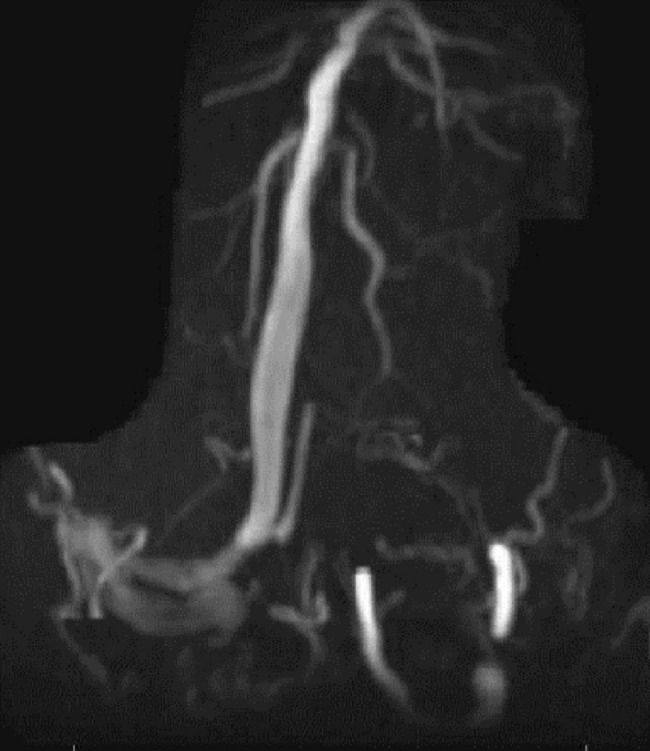
Concomitant MRV Concomitant MRV in the coronal view demonstrates occluded left transverse sinus.

**Figure 5:**
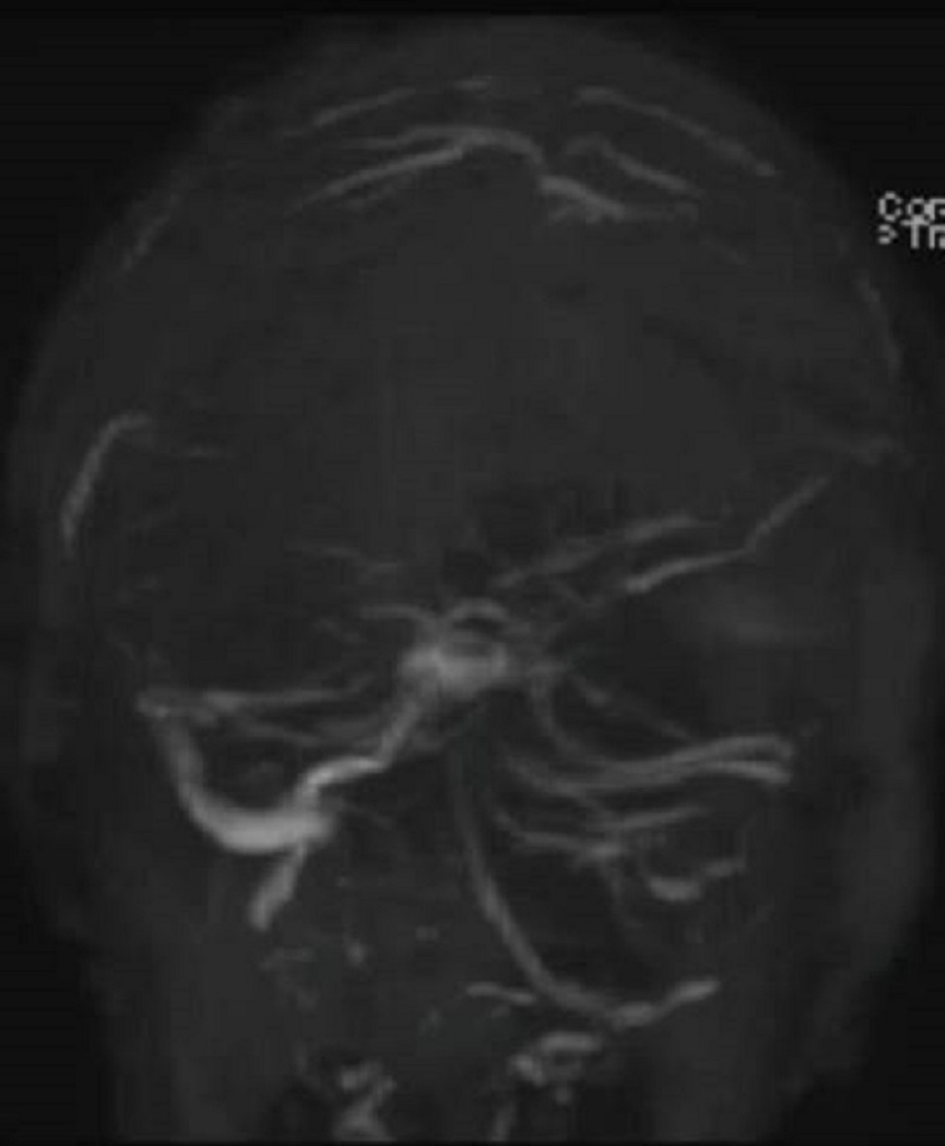
MRV MRV in the coronal view demonstrates near complete occlusion of the entire venous system.

The second patient is a 70-year-old female with a diagnosis of multiple myelomas who presented with a seizure. MRA/V revealed a right temporal venous infarct and an occluded right transverse sinus (Figures 3-5). She was started on a heparin drip. Seven days later, she experienced a neurological decline and required intubation. Repeat imaging revealed occlusion of the superior sagittal sinus, the vein of Galen, the torcula, as well as both transverse sinuses. There was some minor edema in the thalami bilaterally. Given her precipitous decline, it was decided that if any recovery were possible, acute intervention was needed, and waiting for an MRI to rule out thalamic infarction was not feasible.

**Figure 6:**
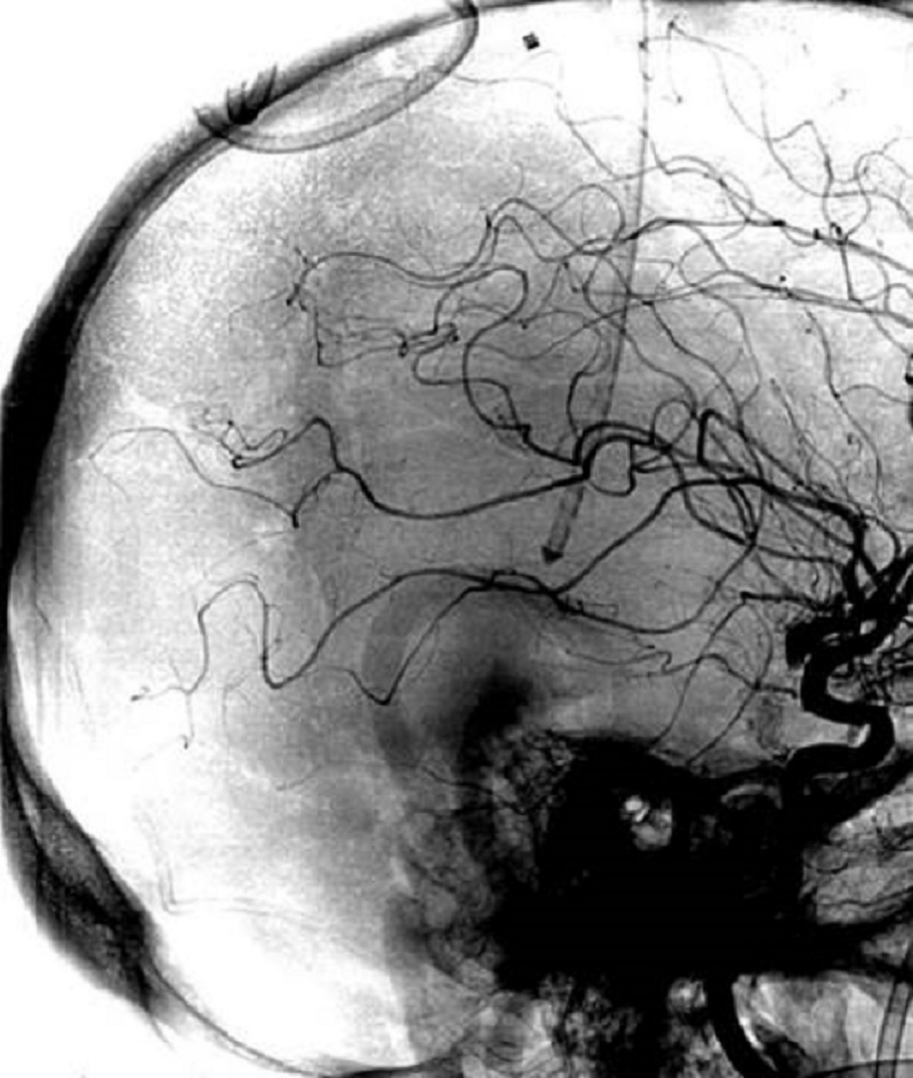
Catheter Angiogram Catheter angiogram, unsubtracted, sagittal plane reveals catheter tip.

**Figure 7:**
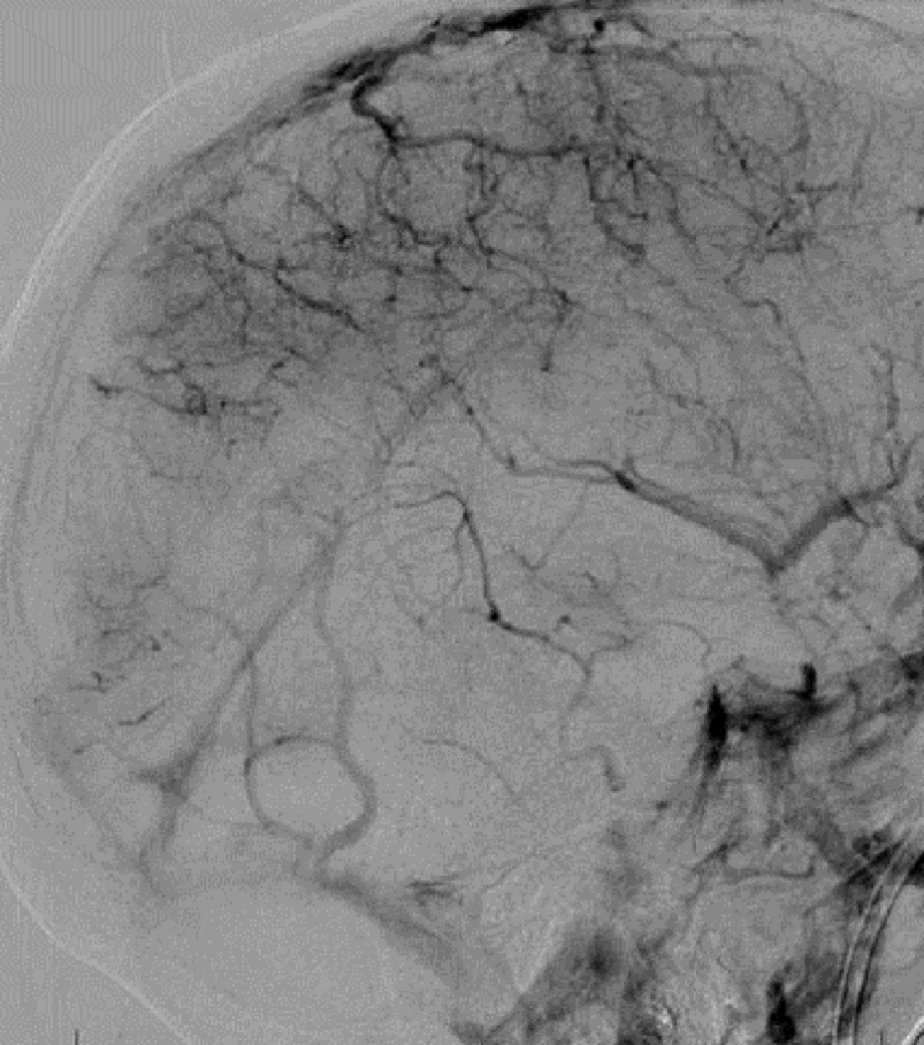
Pre-Treatment Catheter Angiogram Pre-treatment catheter angiogram in the venous phase demonstrates no flow in the distal sagittal sinus, torcula, or transverse sinus, with the beginning of some flow in the sigmoid.

**Figure 8:**
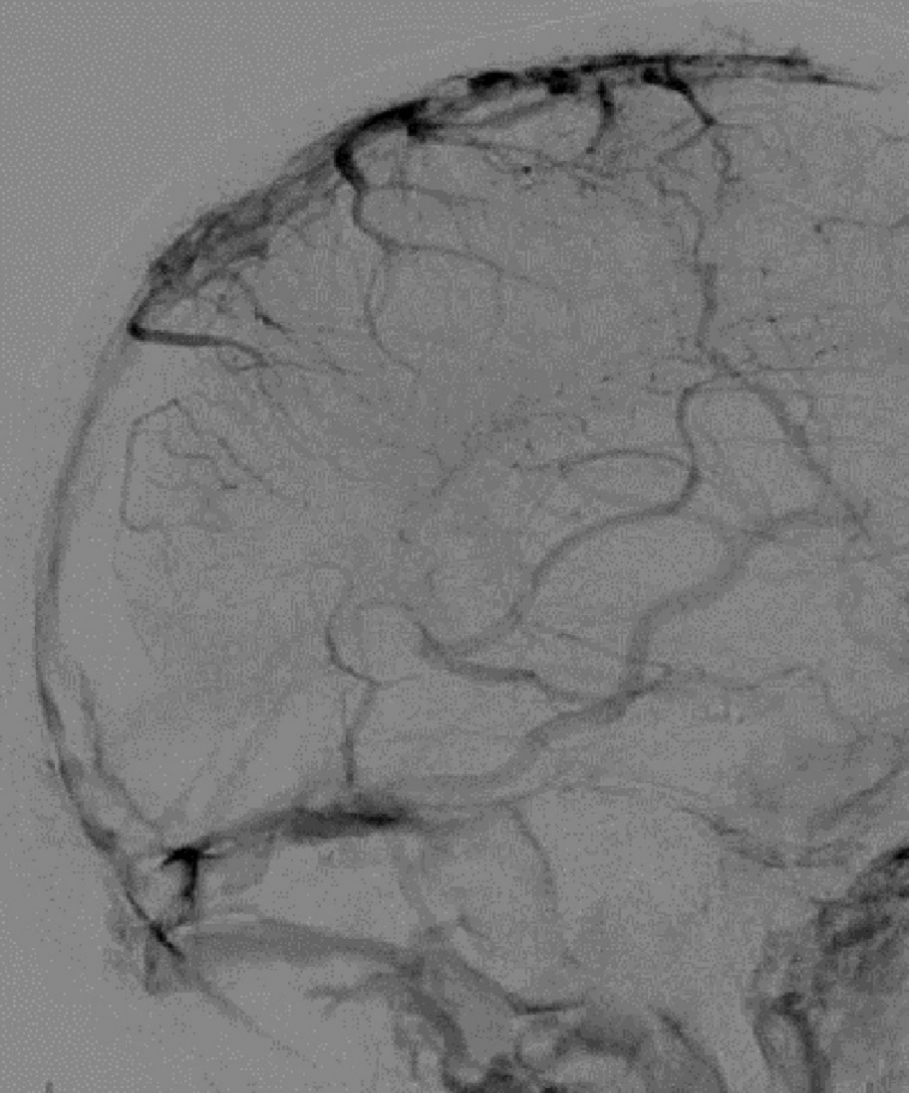
Post-Treatment Catheter Angiogram Post-treatment catheter angiogram in the venous phase reveals restoration of flow in the sagittal sinus, torcula, and transverse sinus, as well as a prominent vein of Labbe.

She was taken to angiography and both stent retriever and aspiration thrombectomy were attempted without recanalization (Figures 6-7). A 0.027-inch microcatheter was placed in the thrombus in the sagittal sinus, and 20 mg of tPA was dissolved in a 1 liter bag of normal saline and infused at 2 mg/hr for ten hours. She was brought back to angiography at the completion of the infusion, which revealed recanalization (Figure 8). MRI confirmed bilateral thalamic infarcts. After twenty-four hours of no improvement in her clinical exam, her family elected to withdraw care.

### Case 3

**Figure 9:**
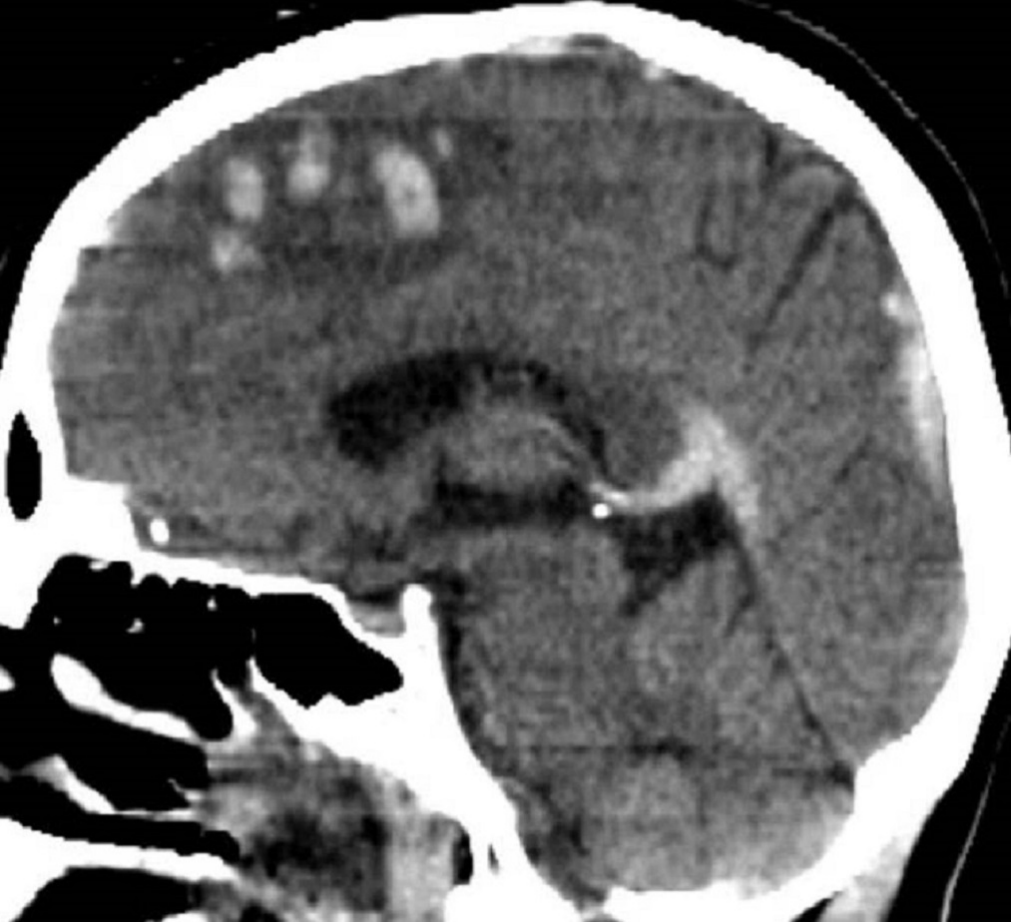
NON-Contrasted CT at Presentation NON-contrasted CT obtained at presentation in the sagittal view reveals hyperdense thrombus in the sagittal sinus, vein of galen, and the straight sinus. A classic appearing venous infarct with hemorrhage and very little mass effect are seen.

**Figure 10:**
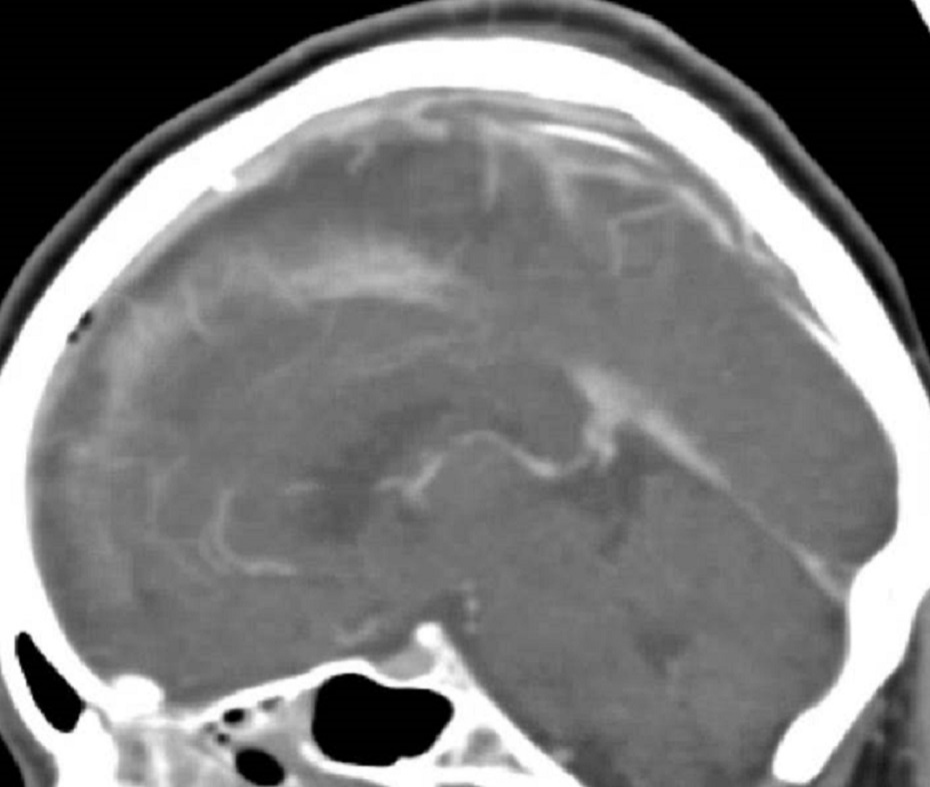
Contrasted CTA 10 hours Post-Infusion CONTRASTED CTA obtained after 10-hour infusion reveals flow through the sagittal and straight sinus, the vein of galen, and the internal cerebral veins. The catheter tip and non-occlusive thrombus in the sinus are visible.

The third patient is a 40-year-old female who presented with a seizure. A CT scan revealed bilateral temporal venous infarcts and superior sagittal sinus, the straight sinus, vein of Galen, and the right transverse sinus (Figure 9). She was intubated for airway protection and started on heparin. On day two, she became hemiplegic with CT evidence of an extending thrombus but no new hemorrhage. She was taken to angiography where both stent retriever and aspiration thrombectomy were attempted without recanalization. A 0.027-inch microcatheter was placed in the anterior third of the sagittal sinus, and 20 mg of tPA was dissolved in a 1 liter bag of normal saline and infused at 2 mg/hr for ten hours. CTA at the completion of the infusion revealed recanalization (Figure 10). She was discharged two weeks later with 3/5 strength on the left upper and lower extremity and was neurologically intact three months later at her follow-up.

## Discussion

The current literature is a dizzying array of small series presenting a large number of techniques with varying degrees of success. In 2009, Rahman et al., published a review of the literature regarding thrombolysis techniques for CSVT [1]. The authors identified 71 manuscripts describing treatment in 161 patients, with a mean of 2.3 patients per manuscript. Approaches included transfemoral, transjugular, and venous sinus puncture via open fontanelle, burr hole, and craniotomy. Drugs included urokinase, streptokinase, and tPA. The dose of tPA varied widely from a minimum of 2 mg/hr x four hours with no bolus, bolus doses ranging from 14 mg to 25 mg, and total doses ranging from 8 mg to 128 mg. 

Rahman et al., also reviewed mechanical thrombectomy and combinations of thrombectomy as well as infusion [1]. The devices included balloon angiography, stenting, Merci, rheolytic catheters, microsnares, and using just a microwire. Haghighi et al., published a review of strictly mechanical techniques in 2014, summarizing the results of 26 manuscripts describing treatment in 64 patients [3]. The treatments included Angiojet, penumbra, ballooning with and without stenting, and microsnare. Since that time, there have been numerous reports using the latest generation of stent retrievers and aspiration catheters [4-5]. These review articles demonstrate the volume of differing techniques; the variety of surgical approaches to treat an individual patient is daunting. Rather than postulating the best techniques, the published literature merely provides a laundry list of what might be technically possible.

Siddiqui et al., retrospectively compared the outcomes of 63 patients who underwent either thrombectomy versus thrombolytic infusion alone at three centers [2]. They found “both treatment options have similar discharge, intermediate and long-term mortality and morbidity. However, device-related complications were more prevalent in the [mechanical thrombectomy] group.” Given the non-randomized, unblinded design of the study, even this modest conclusion is only Level III evidence according to the American Academy of Neurology guidelines. In our cases, we weighed the low quality of available evidence suggesting complications of mechanical thrombectomy versus the benefit of immediate recanalization and avoidance of thrombolytics of patients at risk for hemorrhage; ultimately we decided to attempt these procedures. In our three patients, mechanical thrombectomy was attempted without success or complication. Therefore, the combination of the available literature and our own experience lead us to favor thrombolytic infusion, even in cases with severe neurologic deficits. This may not be surprising as devices optimized for removing arterial clots, approximately 10 mm in length in vessels approximately 4 mm in diameter, struggled with thrombus likely five times that length in vessels almost twice as large.

We waited until all three patients had both clinical and radiographic evidence of decline. Given the relative safety of the intervention as reported in the literature and in our series, this may be too long. Nonetheless, we feel that there is risk to prolong tPA infusion in patients with hemorrhages or at risk for hemorrhage, as CVST patients are. Furthermore, given that initial recanalization is almost always partial, long-term anticoagulation is required even if no clotting disorder is discovered, i.e., IA tPA infusion will not save the patient from long-term treatment with an antiocoagulant. Therefore, it is our current opinion to not treat every patient who presents or whose clinical decline is limited to worsening headaches. That being said, we now perform a follow-up, non-invasive venous imaging before discharge so that we can identify failures of medical management before the patients become critically ill or unconscious.

While we hope this manuscript is helpful in guidance to surgeons with selections of catheters, dose, rate, and duration of infusion, many aspects of the treatment decision remain fairly opaque. This manuscript does nothing to elucidate what constitutes a failure of heparin, what patients are likely to experience such a failure, and what is the proper timing for intervening to maximize good outcomes. A randomized trial is unlikely due to small numbers. Hopefully, further shared clinical experience can help clarify these points.

## Conclusions

In our experience, CVST can be safely, effectively, and reliably recanalized by placing a 0.027-inch microcatheter at the proximal portion of the thrombus and infusing 20 mg of alteplase dissolved in 1 liter of normal saline and infused at 100 ml per hour for an infusion of 2 mg of alteplase per hour for ten hours. By identifying treatment pitfalls as well as our positive results, we hope to provide more specific clinical guidance for surgeons faced with CVST refractory for medical management. Furthermore, we hope that this review and technical notes will spur further discussion and perhaps begin the process of creating a consensus among surgeons who treat this condition.

## References

[1] Rahman M, Velat GJ, Hoh BL, Mocco J (2009). Direct thrombolysis for cerebral venous sinus thrombosis. Neurosurg Focus.

[2] Siddiqui FM, Banerjee C, Zuurbier SM (2014). Mechanical thrombectomy versus intrasinus thrombolysis for cerebral venous sinus thrombosis: a non-randomized comparison. Interv Neuroradiol.

[3] Borhani Haghighi A, Mahmoodi M, Edgell RC (2014). Mechanical thrombectomy for cerebral venous sinus thrombosis: a comprehensive literature review. Clin Appl Thromb Hemost.

[4] Mascitelli JR, Pain M, Zarzour HK, Baxter P, Ghatan S, Mocco J (2015). Sinus thrombectomy for purulent cerebral venous sinus thrombosis utilizing a novel combination of the Trevo stent retriever and the Penumbra ACE aspiration catheter: the stent anchor with mobile aspiration technique. BMJ Case Rep.

[5] Shaikh H, Pukenas BA, McIntosh A, Licht D, Hurst RW (2015). Combined use of Solitaire FR and Penumbra devices for endovascular treatment of cerebral venous sinus thrombosis in a child. J Neurointerv Surg.

